# Barriers to accessing mental health services in The Gambia: patients’/family members’ perspectives

**DOI:** 10.1192/bji.2021.26

**Published:** 2022-05

**Authors:** Lamin F. M. Barrow, Ann Faerden

**Affiliations:** 1 Institute of Health and Society, University of Oslo, Norway. Email: laminfmbarrow@gmail.com; 2 Clinic of Mental Health and Addiction, Oslo University Hospital, Norway

**Keywords:** Barriers, access, mental health, service users, The Gambia

## Abstract

This paper concerns mental health services in The Gambia. It describes local concepts, experiences and knowledge about mental illness and the implications of such beliefs and attitudes for access to mental health services. A pretested questionnaire and interview guide were administered to a sample of patients/family members. Barriers to accessing mental health services were identified. These included beliefs about the causes of mental illness; family decision-making; the scarcity and high cost of services. Obtaining access to mental health services in The Gambia is currently challenging. Importantly, increased community and family education about the causes and treatment of mental illnesses will be required to address these issues.

## Introduction

Effective management of mental illnesses is a major unmet need in many low- and middle-income countries.^1,2^ These illnesses contribute to the costs of health and social care. They are associated with a poor quality of life, loss of employment and increased risk of disability and early mortality.^3^

The Gambia is a low-income country, the smallest within mainland Africa, with a per capita gross domestic product of about US$300^4^ and a population of about 2 million people. Agriculture, tourism and remittances (from a re-export trade) are the main pillars of the economy. The key social challenges the country is faced with include poverty and limited access to jobs. World Bank data^5^ indicate that over 10% of the population live below the poverty line of less than US$1.9 per day. Thirty per cent of adults have not completed primary education. The unemployment rate stands at 22%, affecting mostly young people. This influences many of them to undertake a journey that leads to illegal migration into Europe, looking for greener pastures.

It has been estimated that about 120 000 of the population are affected by mental illness,^6^ but 90% of these people do not access mental health services for their conditions. There is just one psychiatric hospital (Tanka Tanka) and one psychiatric out-patient clinic serving the entire population. These facilities are only found in and around the capital. The only other mental health service is the community mental health team that visits health facilities outside urban areas, according to a planned schedule. Although it is the only service available to most of the rural population, a community team has recently been provided by a few health facilities in urban areas too. Nevertheless, most rural and urban areas are without any mental health service. This situation prevails even though The Gambia has a mental health policy and a strategic plan that seeks to improve access to mental healthcare for its population.

## Method

### Study setting

This study was conducted in the Brikama local government area of The Gambia, which lies in the west of the country, about 40 km south-east of the capital Banjul. It is one of the fastest growing regions, accounting for 37.2% of the national population. The region is partly urban and partly rural. Although there are no recent data about the prevalence of mental illness in the region, the recent population increase and urbanisation might have influenced the risk of mental illness in this population.

### Study design

Convenience and purposive sampling methods were used to identify participants for focus group discussions (FGDs) and in-depth interviews respectively. For the FGDs, participants were identified from five selected communities, with the aim of obtaining a good representation of ages and ethnic groups. For in-depth interviews, patients/family members were invited to participate in the study at mental health service delivery points. Two interview schedules were developed and pretested before they were used for the data collection. A tape recorder was used to record all FGDs and in-depth interviews. Fifteen individuals, between 18 and 54 years of age, participated in the in-depth interviews. Participants in the focus groups were between 18 and 70 years of age. Two of the FGD groups were female and the other three were male. The groups were representative of the main ethnic groups from the region. Thematic analysis was used to analyse the data, which were first translated into English. The transcripts were read several times to gain an overall sense of the themes covered by the interviews and focus groups. This initial analysis was followed by thorough reading to identify different categories and subcategories of issues, which were then further grouped together according to their similarities. A coding scheme that best defined the key themes was created to provide a way of organising the data for further analysis, using colour codes.^7^

This study received ethical approval from The Gambia Government/Medical Research Council joint scientific ethics committee (reference number RO 15 006), but was exempted from review by the Regional Committees for Medical and Health Research Ethics (REK) in Norway (reference number 2015/1002). Written or verbal informed consent was obtained from all the study participants.

## Results

Our analysis of the interview material and issues raised by the focus groups revealed four main factors that acted as barriers to people who wished or needed to access mental health services.

First, family decision-making was of great importance. The decision to go for treatment, where to go and when to go was seen as the responsibility of the family. All the participants interviewed stated that it was primarily their family that decided whether they should seek treatment and the family decided where treatment should be sought. One respondent answered,

‘All the places I visited; it was my family who took me there. You know I am sick; I cannot make decisions. They just ask me to go with them for treatment, but they provide the money’.

Such decision-making about seeking treatment is premised on a widespread belief that it is the family that should take responsibility for a sick family member:

‘if a family member becomes mentally ill, the family takes his responsibility, including seeking treatment for him, because he cannot make decisions and may not have the resources to pay for treatment’.

The second most important issue concerns common beliefs about the causes of mental illness among the interviewees. We were told that these included bad winds, evil spirits, poverty, the use of cannabis, an unmet desire to travel in Europe and childbirth. Participants who believed that mental illness is caused by bad winds and evil spirits are unlikely to come for treatment by psychiatrists whose belief system is based on biomedical foundations. Participants who believed mental illness is caused by poverty and an unmet desire to travel believed that they do not require treatment but a change of situation, although those whose mental disorder is caused by the use of cannabis can be treated by a biomedical approach. As a result of widespread beliefs about the causes of mental illness in this population, treatment seeking is often delayed:

‘when the illness started, my brother suggested that they should take me to the psychiatric hospital, but some other family members said no, they should not because that will worsen my condition as the sickness is cause by bad winds. So, they took me to a traditional healer’.

However, a couple of participants had come to realise that some types of mental illness do require biomedical treatment. One man explained that his son had become sick; he was told not to take him to a psychiatrist because the sickness was caused by evil spirits. Eventually, he did take his son to the psychiatric clinic:

‘I took him to Tanka Tanka psychiatric hospital and he improved, even though I was told that I should not take him there. Now the problem is that I have to buy medication for him every month which is very expensive and not available here’.

The high cost of treatment was identified as a major hurdle by families wishing to access both traditional healers and biomedical mental health services. All the participants had had some experience with traditional treatment. More than half of them said they could no longer access treatment from some traditional healers owing to the high cost:

‘the traditional healer said I should pay eight thousand dalasi [US$187] for treatment, but I have to pay half of the sum before he can start treatment. I could not pay that money at that time, so we left’.

Note that half of employees in The Gambia earn less than 10 000 dalasi per month, so this would have represented 1 month salary. Our participants also highlighted the daily and monthly cost of drugs and injections they needed:

‘Every month, I take one injection and one tablet a day, sometimes it costs 400 to 500 dalasi [US$9–12], the price fluctuates, and sometimes it is not available here, I have to go to Kombo [on the far west coast] to buy it’.

Finally, most participants reported great difficulty trying to access mental health services. Access is limited by the lack of services in most areas and by the very long distances people must travel to service points. One participant, whose son became mentally ill, explained:

‘I could not take my son for treatment because the service was not available […] I was told if I wanted treatment for my son, I must take him to Banjul or Tanka Tanka Psychiatric Hospital’.

Tanka Tanka Psychiatric Hospital is the only psychiatric in-patient facility, located in the western region of the country. It was built in 2009 by a Dutch non-governmental organisation (NGO), Tanka Tanka Foundation, on land donated by the President of The Gambia. It is funded by government subvention, with the assistance of NGO donations.^8^

## Discussion

Our analysis has emphasised that Gambian families are supportive, as they provide the cost of treatment and care for the mentally ill. However, we also discovered that it is the family that decides whether someone with a mental disorder goes for treatment, as well as where they go for treatment. Their decision is often premised on their belief about the putative cause of the illness. There was a plurality of beliefs about the causes of mental illnesses among participants, some of which were in line with modern scientific beliefs, whereas others were not. A similar finding was reported from a similar study in Burundi.^9^ Family decision-making about the perceived cause of mental illness is problematic as it can either delay care seeking or lead to no care being provided at all, because of an assumption that the condition will go away spontaneously. The scarcity of mental health services in the Gambia^8^ hinders individuals from accessing services, as they would either have to travel to the city or use alternative services that are traditional in character. This finding is consistent with findings reported from Nigeria and Ghana,^10,11^ where mental health services are also significantly skewed in favour of urban populations. The scarcity of modern mental health services contributes to the continuing use of traditional and alternative healers, which is a common phenomenon in other African countries too.^12^ A further problem is the high cost of both traditional and biomedical services. That cost deters the mentally ill from accessing services. In a country where a typical salary per year is just US$1000 after tax, our informants reported that traditional healers are reported to charge up to US$187 for treatment, and biomedical daily medication and injection cost about US$9–12, depending on the geographic location. This is one of the reasons for such a diversity of mental healthcare, a phenomenon that has also been reported in other African countries, including Nigeria, South Africa and Tanzania.^13–15^

### Implications

Access to mental healthcare is not only about people who are mentally ill. It has implications for the family, wider society and the government too. Improving access to mental health services should take these broader socio-cultural and political factors into account. Biomedical services for mental healthcare should be available at all major health centres and hospitals. There should be easier access to mental health services, as well as improved community and family education on the causes and treatment of mental illnesses, which would improve utilisation of services.

## Data Availability

Data supporting the findings of this study are available from the corresponding author on request.
